# Editorial: Neighborhoods and community: the role of built and social environment for healthy aging

**DOI:** 10.3389/fpubh.2026.1883724

**Published:** 2026-06-17

**Authors:** Sojung Park, Amanda Lehning

**Affiliations:** 1 Brown School, Washington University in St. Louis, St. Louis, MO, United States; 2 University of Maryland Baltimore, Baltimore, MD, United States

**Keywords:** age-friendly community, aging in place, built environment, digital environment, healthy aging, neighborhood, social environment

Population aging represents one of the most significant demographic and public health transformations of the 21st century. By 2050, the global population aged 60 years and older is projected to reach 2.1 billion (1). Alongside this demographic shift, there has been growing recognition that neighborhood and community contexts influence mobility, caregiving, participation, health, and quality of life in later life. Research across gerontology, public health, social work, environmental psychology, and urban planning has emphasized the interaction between older adults and their physical and social surroundings, from foundational work in environmental gerontology to more recent scholarship on age-friendly communities, aging in place, and environmental inequality (2–6). Yet aging in place is not inherently beneficial; the capacity to age well within community is shaped by affordability, accessibility, safety, social connectedness, and broader structural inequities. Although framed around the built and social environment, the 10 contributions in this special issue also extend that framing into digital, institutional, and governance dimensions. We organize our reflections around three themes.

## Theme 1: neighborhood contexts continue to matter in later life

Several contributions reaffirm that neighborhood conditions remain central to later-life health and wellbeing while complicating the assumption that proximity automatically confers protection. Nouri and Chaudhury examine built environment factors influencing outdoor walking among people living with dementia in Metro Vancouver, finding that transit access, mixed land use, sidewalk suitability, and pedestrian-oriented design jointly supported mobility and engagement. Miao et al. similarly demonstrate that older adults' experiences of community sports parks extend beyond physical activity to include accessibility, comfort, sociability, safety, and intergenerational engagement.

Other studies highlight how neighborhood conditions may either support or undermine health and quality of life in older adulthood. Choi et al. report that greater neighborhood social cohesion was associated with lower psychological distress among caregiving spouses, particularly among those lacking nearby social support networks. Versey et al. show that housing-cost concerns and frequent residential moves were associated with poorer self-rated health and higher substance use among older adults living in New York City NORCs, settings often presumed stable. Together, these studies illustrate neighborhoods as potential sources of mobility, connection, stress, exclusion, or financial precarity in later life. More broadly, they reinforce aging in place not as the goal itself, but as a precondition for autonomy, participation, and quality of life.

## Theme 2: expanding the definition of environment

A second cluster broadens the concept of environment beyond physical space and proximate social ties. Golant's conceptual ELDERDES model argues that older adults' homes increasingly function as digital environments through the integration of telehealth, monitoring systems, virtual services, and AI-assisted technologies. The paper also introduces digital destinations as an emerging dimension of environmental gerontology and suggests that digital environments may become an increasingly important arena for research and intervention alongside the built and social environment.

Other contributions expand the concept of environment through institutional and governance perspectives. Tan and Huang compare gated retirement and open multigenerational communities in China, finding that age-segregated models may reinforce spatial inequality and exclusion, while more integrated community forms demonstrate stronger vitality and age-friendliness. Wu et al. examine age-friendliness in urban fringe communities through a resilience-informed framework, emphasizing how community space, services, social interaction, and participation jointly shape aging experiences in rapidly changing urban settings. Together, these studies suggest that age-friendly environments are not simply designed spaces, but socially and politically constructed systems shaped by governance decisions, institutional priorities, and patterns of inclusion and exclusion.

## Theme 3: building supportive environments for healthy aging

A third cluster shifts attention to implementation and the often-invisible labor of building and sustaining environments that support healthy aging. Greenfield's reflective case study of launching the Glen Rock Neighborhood Network illustrates how human, social, and financial capital were mobilized during the start-up phases of a Village initiative, an area that has received less attention than research on mature programs. Chan et al., applying the Consolidated Framework for Implementation Research to Singapore's Active Aging Centers, identify both facilitators and barriers to implementation, including strong policy support and community partnerships alongside tensions between centralized governance and local flexibility. Their findings also highlight ground-up innovations developed by frontline staff to improve engagement and sustainability.

Koren et al. add a research-infrastructure perspective through the Healthy Aging Initiative; a longitudinal interdisciplinary cohort study embedded in senior housing communities. Across these studies, supportive environments emerge not as static features of communities, but as ongoing and resource-intensive processes shaped by funding, staffing, governance structures, and local priorities.

## Looking ahead

Several directions emerge from this collection. First, the contributions highlight the importance of broader geographic and methodological representation, including work from Latin America, Africa, South Asia, rural communities, and Indigenous contexts. Second, they suggest the value of examining differentiated community forms rather than treating “age-friendly community” as a single object of study. Finally, the issue points toward continued attention to digital and layered social environments, implementation processes, frontline-staff perspectives, and the direct voices of older adults and care partners themselves.

## Conclusion

**Figure 1:**
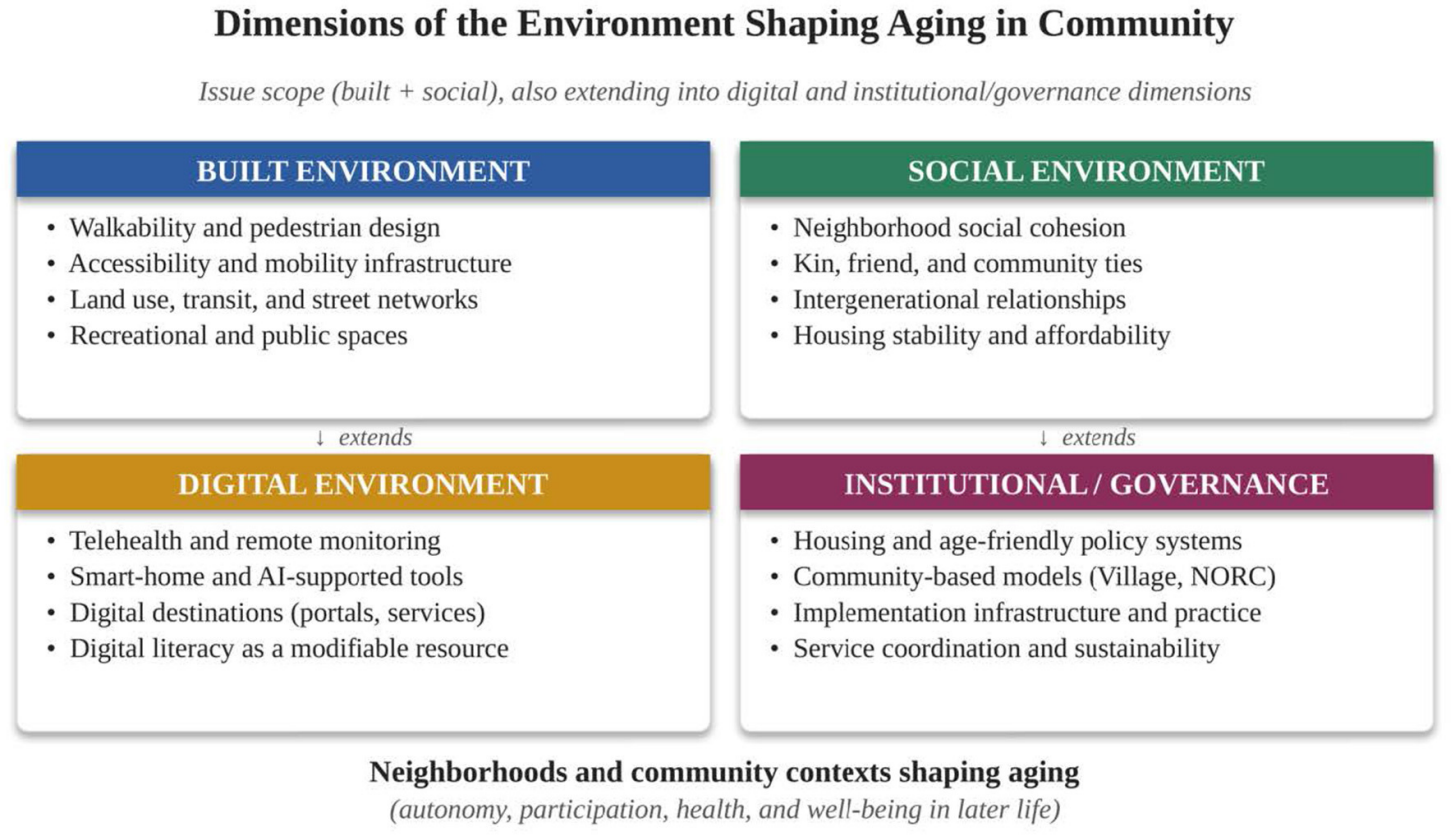
A conceptual schematic of four environmental dimensions shaping aging in community. The special issue is framed around the built and social environment **(Top row)**; the contributions also extend the conversation into digital and institutional/governance dimensions **(Bottom row)**. Each dimension is illustrated with representative concepts surfaced by work in the field.

The articles in this special issue point to an important shift in healthy aging research and practice (see Figure 1). While neighborhoods and communities remain central to later-life health and wellbeing, the environments shaping aging are becoming increasingly multidimensional, extending beyond physical spaces to include digital infrastructures, institutional systems, and governance structures. Future scholarships should continue moving beyond questions of whether older adults can remain in their homes and communities toward deeper consideration of what kinds of environments enable people to age with autonomy, connection, safety, and dignity. As these contributions suggest, supportive environments are not static features of place, but evolving systems shaped through policy decisions, technological change, and collective community action.
